# Transportation and Access to Healthcare in Morocco: An Exploratory Study of Guelmim-Oued Noun Region

**DOI:** 10.5334/aogh.4063

**Published:** 2024-02-09

**Authors:** Jamal Tikouk, Asmaa Ait Boubkr

**Affiliations:** 1 Applied Modeling in Economics and Management Laboratory, University of Hassan II Casablanca, Casablanca, Morocco

**Keywords:** canonical correlation, transportation, barriers, access to healthcare

## Abstract

**Objective:**

The aim of this study was to examine the correlation between accessibility to healthcare facilities and transportation in the Guelmim Oued Noun region of Morocco, where transportation barriers continue to pose a major challenge to accessing healthcare, despite efforts aimed at reducing access barriers.

**Methods:**

Data collection for this study involved the administration of a survey among 328 outpatients residing in the Guelmim Oued Noun region, Morocco. The utilization of canonical correlation served as the analytical method, employed to quantify and assess the relationship between transportation related barriers and the access of healthcare services in the specified region.

**Results:**

Our research reveals that transportation factors account for approximately 25% of the variation in access to healthcare services. The number of transportation modes utilized by outpatients and the affordability of transportation were found to be significant contributors to the transportation dimension. These findings confirm the significant relationship between transportation and access to healthcare facilities in the region under investigation.

**Conclusion:**

Further research is recommended to specifically address transportation barriers to healthcare access services among socially excluded populations, with a focus on promoting mobility inclusivity.

## 1. Introduction

Based on recent statistics from the World Bank and World Health Organization (WHO) research, half of the world’s population declared having difficulty reaching health services [1]. According to the broad literature, transportation has been recognized as a key factor in having access to healthcare facilities, particularly in developing countries where access to suitable transport with affordable cost remains a real challenge [2,3,4,5,6]. Recently, the Moroccan government has made noticeable efforts to enhance access to health services for the Moroccan population [7]. Nevertheless, the situation remains a serious issue and an elusive objective for public policymakers [7]. In this regard, a large body of literature has been published with respect to the determinants of access to health services, namely age [8], gender [9], medical coverage, and chronic diseases [8]. The literature on social determinants of health, particularly in regards to healthcare access, has extensively addressed issues such as poverty, education, and language barriers. However, there has been a lack of research specifically examining the link between transportation disadvantage and healthcare access. Despite the recognized importance of transportation as a determinant of health, there is a dearth of studies addressing the extent to which transportation barriers prevent individuals from accessing healthcare. Furthermore, official statistics on the number of patients who postpone or cancel appointments due to transportation issues, as well as the impact of these barriers on healthcare outcomes, are scarce.

Access to health care is a complex and multidimensional concept. The United States Institute of Medicine (IOM) defines access to care as the timely use of health services by individuals to achieve the best possible health outcome [10]. In the same conceptualization, Waters reduces this concept of access to care to the concept of health care utilization [11]. The term utilization is defined by some authors as the result of the interaction between the behavior of the individual who uses healthcare and the behavior of the professional who guides the individual through the system; it is the institutional response to the needs of the individual’s expressed demand [12]. Frenk and White define access to care according to the characteristics of the population. Thus, for these authors, access to care refers to the capacity of the population to seek and obtain care [12].

It will be a reasonable starting point to define a theoretical framework. For this purpose, there is a wide range of explanatory framework identifying factors related to health care access. In this regard, Andersen’s Socio-Behavioral Model [13] is one of the most comprehensive and frequently used models. The earlier version of this model was developed in 1960 before being subject to many changes over time. Recent studies have moved from an individual-level focus to covering other factors related to the external environment and the healthcare system. The model used in this research identifies three main determinants of healthcare services: predisposing factors, enabling factors, and perceived needs:

- The predisposing factors: refer to the socio-demographic dimensions, such as education, age, gender, marital status, race/ethnicity, as well as a set of beliefs.
- The needs factors include individuals with regard to functional capacity, symptoms, diseases, and state of health.
- The enabling factors or resources, include family characteristics such as income, insurance coverage, access to services (transportation and distance to care), and community characteristics such as availability of resources and region of the country.

### 1. Transportation and healthcare access

This section focuses on studies with respect to the link between transportation factors and access to healthcare facilities. Transportation plays a key role in enabling and facilitating access to healthcare services. The majority of studies with regard to healthcare access are still dominated by the focus on need factors rather than enabling factors. We argue that the role of transportation is less studied and little is known about the manner in which travel time, mode of travel, distance, affordability, and availability affect access to healthcare facilities.

#### 1.1. Travel time

Travel time plays a crucial role in determining healthcare access, as evidenced by a survey conducted among caregivers of children visiting urban pediatric clinics. This survey revealed that missed appointments were often attributed to transportation problems, such travel time and transportation cost [14]. Public transport travel time for outpatients is reported as a significant factor for missing kidney dialysis sessions [15]. Additionally, distance was a predictive factor for not attending in-between follow-up appointments (6- and 9-months), whereas it was not predictive for the 12-month or 3-month follow-up appointments following a gastric band being fitted [16]. Another research revealed that public transport travel times were longer for outpatients who did not attend follow-up appointments compared to those who did [17].

#### 1.2. Mode of travel

Beyond studies discussing travel time factors outlined a significant association between health service access and the mode of travel used to reach the clinic for prenatal care. Women who owned private car had greater chances than those who used public transit [18]. By analyzing the preventative health care visits of Latino children in an urban area, a study found that 21% of parents cited transportation problems as the single most important challenge for not bringing their children in for systematic medical examinations [19]. Another study conducted retrospective research based on a sample of 406 cancer outpatients between 2000 and 2007. According to the demographic features of the respondents, the study sought to examine the relationship between commuting mode and the likelihood of receiving first-line chemotherapy. The findings revealed that outpatients who came from communities with a larger number of households without a vehicle had a lower chance of receiving first-line chemotherapy [20]. In the same perspective, walking or taking public transit to reach medical treatment was a significant predictor of not having regular care (Oddo Ratio 1.44). Outpatients who did not use private transportation were also more likely to miss medical appointments (Oddo Ratio 1.45) [21]. A survey among 203 children’s caregivers, showed that 21% of inner-city children encountered transportation difficulties to timely health treatment. The absence of a car was indicated as the particular barrier by 62% of those polled, outnumbering other factors such as excessive distance, price, or difficulty of public transit [19]. Also, having a private car has played a role in keeping appointments based on a study about 183 urban caregivers from Houston. The study showed that the inability to find a ride resulted in at least one missed appointment for 25% of the sample. The study also showed that 82% of those who kept their appointments had access to a car, compared to just 58% of those who did not keep their appointments [22]. Another study performed in rural North Carolina has examined the association between transportation and healthcare utilization for chronic care management. The results showed that outpatients with a driver’s license had 2.29 times more healthcare visits for chronic care and 1.92 times more visits for regular examinations than those who did not. Having family or friends who could provide transportation had 1.58 times more visits for chronic care than those who did not [23].

#### 1.3. Distance (travel distance)

Regarding aspects relative to distance, previous research in developing countries found compelling evidence that geographic proximity to healthcare facilities has a significant impact on primary healthcare use. However, the distance may not completely account for accessibility because transportation and mobility issues also play a major role [24]. On the other hand, rural outpatients reported more transportation problems and longer travel distances to health care providers, as well as a higher burden of travel for health care as measured by distance and time spent [25]. In their research, that did not include urban counterparts, 31% of rural adult human immunodeficiency viruses (HIV) outpatients lacked transportation and 37% missed visits owing to mobility issues. In the same context, the results of a survey conducted in Zambia with 900 participants demonstrated an inverse relationship between distance or travel time to health facilities and the use of health services.

#### 1.4. Affordability (cost)

Importantly, many studies have pointed out the linkage between transportation affordability and access to healthcare facilities. A study used a multivariate logistic regression on outpatients who had free surgery from a non-governmental organization to demonstrate the influence of lowering the transportation cost barrier on surgical usage. Also, the researchers concluded that when transportation costs are covered, the surgical no-show rate is lowered by half [26]. Also, transportation cost is one of the most commonly addressed factors to access to healthcare services, especially for low-income, disabled, elderly, and geographically isolated populations [27]. In their research, based on a semi-structured interview in Ethiopia, the role of the transport cost was also revealed as a determinant of renouncing access to human immunodeficiency virus (HIV) Counseling and Testing, and Antiretroviral Treatment in Ethiopia [28]. A later study explored how travel distance and other transportation barriers are associated with dental utilization in a Medicaid expansion population. The results obtained demonstrated a significant impact of transportation costs on the use of dental services among Medicaid-insured Adults [29].

#### 1.5. Availability of transportation

Consistent with the previous literature related to the availability of transportation. Lack of public transportation has been recognized as a major barrier to healthcare access. The availability of transportation was identified as a barrier for the indigent population.^1^ In the same vein, a qualitative study was performed to assess barriers and facilitators to HIV clinic visit adherence among HIV-positive women in the rural southeastern region of the United States. The results showed that the lack of transportation means was reported as a key barrier behind missed appointments [30]. About 3.6 million Americans miss at least one medical trip a year because of lack of transportation means and that these people are more likely to be older, poorer, female, minority, and less educated [31]. Furthermore, the availability of transportation was significantly associated with the follow-up appointments for outpatients with spinal cord injuries in Birmingham (Alabama) [32]. Another study focusing on access to medical services among people with unmet healthcare needs in Korea reported that 20.9% of the population experienced barriers to receiving necessary medical services due to a lack of transportation access, especially for low-income, disabled, elderly, and geographically isolated populations [33]. Moreover, in a study of Ghana Community-based Health Planning, the availability of transportation was reported as one of the main challenges behind access to maternal and child healthcare services in rural Ghana [34].

A similar study conducted in Malawi has revealed that both availability and affordability of transport can be barriers to delaying access to health care [35]. In the same vein, another study interviewed 25 individuals with disability in rural, northern Namibia and identified lack of transportation, cost of transportation when available, and availability or distance to care as the main barriers [36].

The gap in the literature highlights the need for further research on transportation disadvantages and its impact on healthcare access. The measure of this link is crucial for targeting the most contributing factors to transportation barriers. In this vein, the current research contributes to the extent of the literature by providing empirical insights into the link between transportation barriers and access to healthcare services. Another interesting original contribution of our research is the application of the canonical correlation analysis which is more convenient when the objective it is to measure the link between two sets of variables. Consequently, we surmise that there is a significant correlation between transportation barriers and access to healthcare services in the region studied.

Regarding the purpose of the study and based on prior work [37], we consider that transportation is an enabling factor. If transportation issues related to healthcare access were extensively discussed in developed countries, the problem is less debated in Morocco where more studies are needed to shed light on transportation barriers and provide practical solutions.

## Aims and objectives

This paper is structured into three sections. The first section outlines the theoretical foundation of our study and empirical research related to the link between transportation and access to healthcare. The second section is methodological. It presents data and its source, and the method of analysis adopted in this research. This method is also illustrated through examples. The Third section is where we present the results and discuss the empirical as well as managerial implications of the results.

## 2. Methodology

### 2.1. Study setting

**Figure 1:**
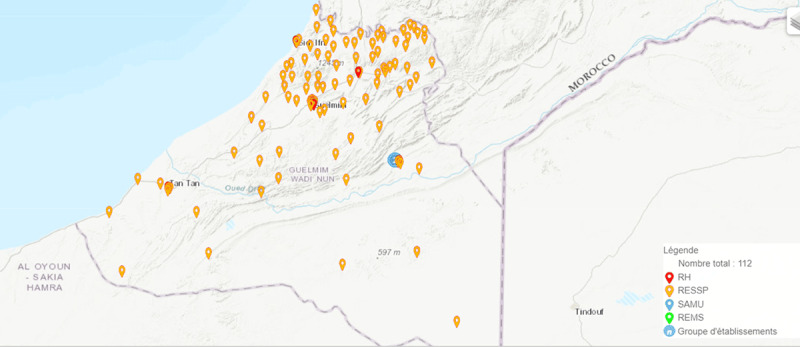
Healthcare facilities in the region Guelmim Oued Noun. Source: Ministery of health and social protection, Morocco.

The Guelmim Oued Noun region is located in Morocco and is one of the twelve regions in the country. It is bordered to the north by the Souss-Massa region, to the east by the Moroccan-Algerian border, to the south by the Laâyoune-Sakia El Hamra region and the Mauritanian border, and to the west by the Atlantic Ocean. The region encompasses four provinces: Guelmim, Tan-Tan, Assa-Zag, and Sidi Ifni, and has 53 communes. The region has an area of 46,108 km^2^ and a population of 433,757, with 35% living in rural areas. The regional healthcare system is divided into four districts and includes five public hospitals with a total of 375 beds. There are 99 healthcare centers in the region, 77 of which are located in rural areas. The ratio of healthcare facilities per inhabitant is about 4,545, and the ratio of inhabitants per public hospital bed is 1,200. The private sector is also a significant player in the region’s healthcare system. As shown in Figure 1, the distribution of healthcare facilities demonstrates the remarkable disparities between provinces in the region. The Time required to access medical specialties for rural population is about a minimum of 30 min and depend on the availability of public transportation with unplanned schedules. The majority of rural territory are socially vulnerable with low socioeconomic status.

### 2.2. Data collection and analysis

For the purpose of our study, a survey based on a questionnaire was conducted to gather data relative to transportation factors that relate to healthcare facilities. The questionnaire was structured in three main parts. The first section focused on demographic characteristics, including gender, age, income, education level, marital status, medical coverage, residence, household size, employment status, presence of chronic conditions, and presence of physical disabilities. The second section addressed variables related to access to healthcare facilities. The third section included transportation factors such as wait time, mode of transportation, travel time, affordability, distance, and number of transportation options before accessing healthcare facilities. The questionnaire was tested with a small sample of 10 outpatients to ensure its validity and to check for comprehension and clarity of questions. The goal was to ensure that the questionnaire did not cause any confusion.

#### 2.2.1. Statistical methods

##### 2.2.1.1. Sample size

The sample size of the current study was computed by using the formula of Cochran [38].


1 \[
{\bf n} = [{\bf p} ({\bf 100 - p})^{*} {\bf z}^{2}]/{\bf e}^{2}
\]

where:

- n is the needed sample size;
- p is the percentage occurrence of a state or condition fixed to 50%;
- e is the percentage maximum error required fixed to 5%;
- z is the value corresponding to the level of confidence required fixed to 1.96.

With these parameters in mind, the minimal sample size required for this study was determined to be 384 observations. The rate of participation in the study was 85% (approximately 328 outpatients). The sample selection was based on a non-probability sampling method, specifically convenience sampling, which is an alternative to probability sampling. This method of sampling is commonly employed in situations where access to the population is restricted by the unavailability of a sampling frame and the reluctance of outpatients to participate in the survey [39].

##### 2.2.1.2. Overview of Canonical Correlation Analysis method

The simple canonical linear analysis is a multivariate statistical technique broadly used for examining relationships between two sets of variables in such a way as to maximize the correlation between each pair [40]. These correlations are commonly known as canonical correlations, and the linear combinations are canonical variates (CV). The idea behind the canonical correlation Analysis technique is to create a number of canonical variates, each consisting of a linear combination of one set of variables (X_i_) in the following equation:


2 \[{\bf{C}}{{\bf{V}}_{{\bf{xi}}}} = {{\bf{a}}_{{\bf{i1}}}}{{\bf{X}}_{\bf{1}}} + {{\bf{a}}_{{\bf{i2}}}}{{\bf{X}}_{\bf{2}}} + \; \cdots + {{\bf{a}}_{{\bf{iP}}}}{{\bf{X}}_{\bf{P}}}\]

Additionally, a linear combination of the other set of variables (Yi) has the following equation:


3 \[{\bf{C}}{{\bf{V}}_{{\bf{yi}}}} = {{\bf{b}}_{{\bf{i1}}}}{{\bf{Y}}_{\bf{1}}} + {{\bf{b}}_{{\bf{i2}}}}{{\bf{Y}}_{\bf{2}}} + \; \cdots + {{\bf{b}}_{{\bf{iP}}}}{{\bf{Y}}_{\bf{P}}}\]

The goal of canonical correlation Analysis is to estimate the parameters, or canonical weights (a_ij_ and b_ij_), which maximize the function of the correlation between canonical variates noted CV_Xi_ and CV_Yi_. The first canonical correlation, Corr. (CV_Xi_, CV_Yi_), is the strongest possible correlation between a linear combination of variables in the exposure set and a linear combination of variables in the outcome set. In the context of this study, canonical correlation analysis has several advantages over regression analysis [41]. For example, it permits the specification of more than one dependent variable and is more flexible in that both dependent and explanatory variables can be either metric and/or nonparametric.

#### 2.2.2. Data analysis

All data analyses were conducted using the Statistical Package for the Social Sciences (SPSS) version 25.0 for Windows. Descriptive statistics were calculated for all items, including measures of distribution, central tendency, and variation as appropriate. Moreover, Spearman’s Rank Correlation analyses were conducted to determine the strength of the correlation between two sets of variables. Significance and high significance levels were set at P < 0.05 and P < 0.01 respectively.

#### 2.2.3. Independent and dependent variables

##### 2.2.3.1 Dependent variables

In this study, we define “access to healthcare” as the actual utilization of health services in order to achieve optimal health outcomes. The most commonly used theoretical model for examining access to healthcare is the Andersen Model [42]. There are various ways to measure access to healthcare services, however, there is currently no consensus in the literature on the best approach [43]. In this study, we assess perceived access to healthcare by asking respondents two questions:

- The first question is: “How many times have you visited the healthcare structure during the last 12 months?” The response to this question is a discrete variable.
- The second question is related to the difficulty in accessing healthcare services during the Covid–19 pandemic, this was approached by an ordinal question: “Can you rate the difficulty in accessing healthcare services during the Covid-19 pandemic?” The answer to this question is a Likert scale that ranges from 1 to 5 points.

##### 2.2.3.2 Independent variables

Based on the understanding of existing literature and theories, this study initially considered a wide array of variables, as shown in Table 1, to approach transportation barriers. Five variables have been retained.

**Table 1:** Variables Approaching Transportation Factors.


GROUPE 2:	VARIABLES		REFERENCES

**Transportations** **Factors**	Travel Distance Km: (Continuous)	The length covered between two points or locations during a journey or transportation activity, specifically between residence areas and healthcare facilities. It is typically measured in units such as miles or kilometers	[31] [44] [45] [46,47]

	Availability of transportation: (Liker scale of 5 points)	The extent or degree to which various modes of transportation are accessible and ready for use within the region, measured by a Likert scale.	

	Number of Transportation Modes: (Discrete)	The count or quantity of different modes of transportation available for use within a given system or network in the region of Guelmim Oued Noun	

	Cost of Transportation (MAD): (Continous)	The monetary expense associated with utilizing various modes of transportation for reaching healthcare facilities.	

	Waiting Time (min) (Continuous)	The amount of time an outpatient spends waiting for and anticipating the arrival of transportation mode	


## 3. Results and Discussion

As previously mentioned, a total of 328 outpatients responded to the branched survey, which asked participants to respond to different questions relative to healthcare access and transportation barriers. A socio-demographic information was collected from all outpatients as part of the current study as reported in Table 2.

**Table 2:** Sample Characteristic (n = 328).


VARIABLES		CATEGORY	COUNT (N = 328)	FREQUENCY (%)

Gender		Female	173	52.7%

		Male	155	47.3%

Age (Years) Median	[Q1; Q3]		37 [28–52 years]	

Size Household Median	[Q1; Q3]		4 [3–6 persons]	

Matrimonial Status	Single		89	27.1%

	Divorced		14	4.3%

	Married		199	60.7%

	Widowed		26	7.9%

Level of instruction	Analphabet		88	26.8%

	Coranic school		45	13.7%

	Primary school		68	20.7%

	Secondary school		74	22.6%

	University		53	16.2%

Employment Status	Employed		85	25.9%

	Not employed		243	74.1%

Residence Area	Suburban		26	8.0%

	Rural		162	49.5%

	Urban		139	42.5%

Medical Coverage	Obligatory insurance Disease		35	10.7%

	Others		18	5.5%

	Private insurance		14	4.3%

	MASSEU [26]		173	52.7%

	Without		88	26.8%

Monthly Income	Without		46	14.0%

	Less than 2000 Dhs		156	47.6%

	2000–3000 Dhs		45	13.7%

	3000–5000 Dhs		59	18.0%

	More than 5000 Dhs		22	6.7%

Level life Perception	Very low		28	8.6%

	Low		76	23.3%

	Average		180	55.2%

	Good		42	12.9%

Percieved Difficulty accessing transportation	Yes		184	56.1%

	No		144	43.9%


### 3.1. Sample Characteristics

The analysis performed over 328 outpatients revealed that 52.7% of the study population was female. The median age of the sample was 37 years. The average household size was four persons. The most common area of residence among the participants was 49.5% rural. 60.7% of the sample was married. Those who are unemployed represented 74.1%. Also, 74% of outpatients reported having medical coverage. The proportion of instructed outpatients was 73.2%. Overall, the sample studied of outpatients who had no income constituted 14.0% of the sample, and 47.6% were under 2000 DHS per month. Outpatients whose level of perception is under or equal to the average is about 71%.

**Figure 2:**
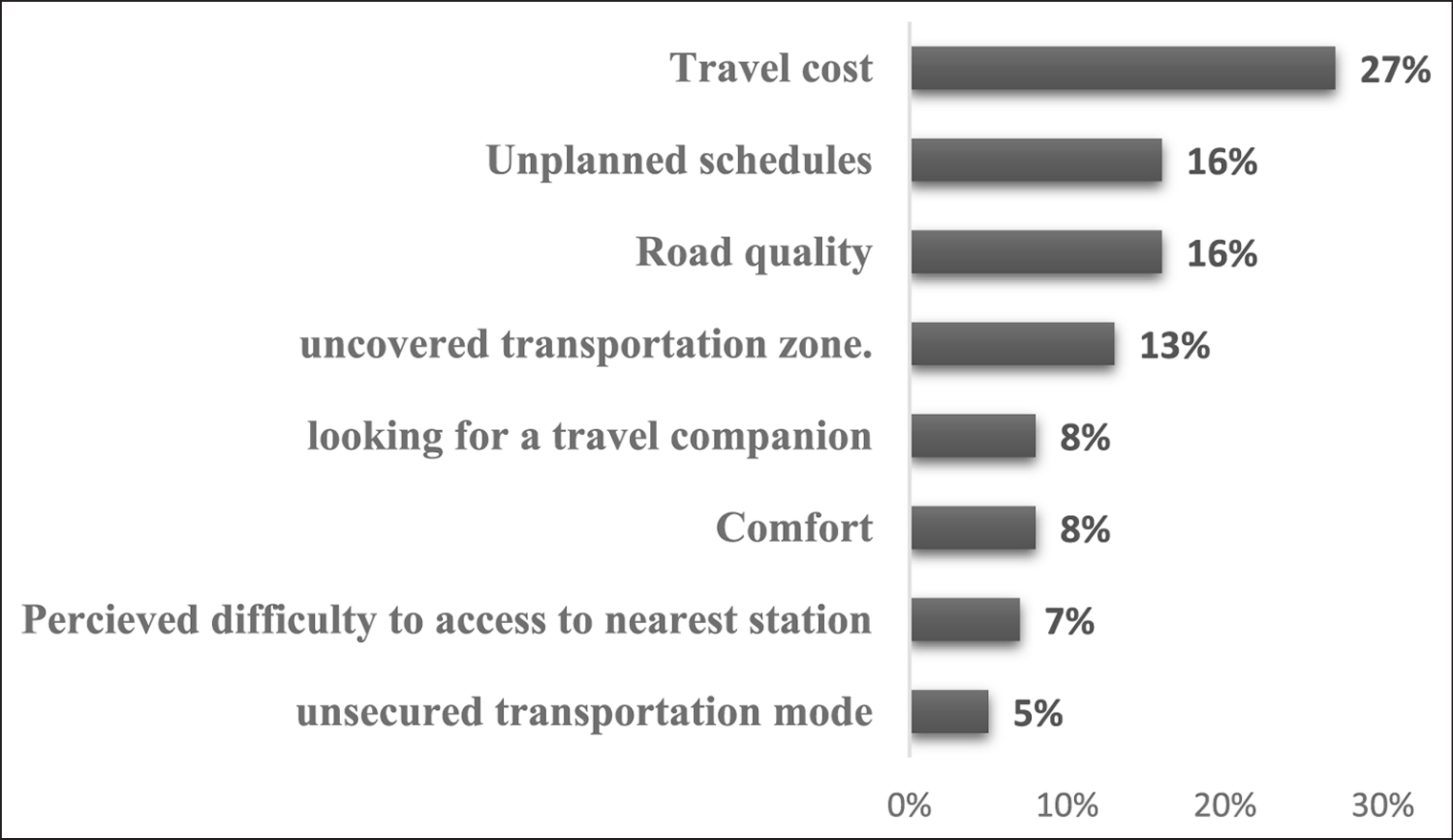
Transportation barriers most experienced by outpatients.

Figure 2 indicates that around 56.1% of outpatients seeking healthcare services faced transportation-related obstacles. This was particularly evident during the Covid-19 pandemic, a time when healthcare authorities implemented numerous measures to curb the virus’s spread, which in turn impacted access to healthcare services due to transportation barriers [48]. The most common transportation barriers were travel cost (27%), Road quality (16%), Unsuitable schedules (13%), and underserved areas with a percentage of 13%. These barriers were commonly encountered in rural areas where the road network was denser which increased the number of transportation modes in order to gain the nearest Bus or Taxi station.

#### Bivariate analysis: Correlation analysis

The primary objective of this correlation analysis is to explore the correlation coefficients between difficulties in accessing healthcare facilities (Y1) and the number of medical visits (Y2), along with various transportation barriers, including Travel Distance, Availability of transportation, Number of Transportation Modes, Cost of Transportation, and Waiting Time. The analysis employs the Kendall-tau correlation coefficient to evaluate the strength of the linear relationship between transportation barriers and access to healthcare services. The overarching goal is to understand how these transportation factors are associated with healthcare access. Notably, as outlined in Table 3, the findings highlight a significant correlation, specifically pointing to the relationship between the number of transportation modes taken and the affordability of transportation.

**Table 3:** Matrix Correlation Between Transportation Related Barriers and Access to Healthcare Facilities.


VARIABLES	Y1	Y2	X1	X2	X3	X4	X5	X6

Difficult to access to healthcare services (Y1)	1							

Number of medical visits (Y2)	0.07	1						

Mode of transportation (X1)	0.07	0.25	1					

Number of mode of transportation (X2)	0.32***	0.17***	0.045***	1				

Waitin time (X3)	–0.09	–0.06	0.07	–0.11**	1			

Level of availability of transportation (X4)	–0.08	–0.07	–0.07	–0.16***	0.04	1		

Travel cost (X5)	0.22 ***	0.21***	0.04***	0.42***	–0.01	–0.21***	1	

Distance (X6)	0.03	–0.03	0	0.28***	–0.01	–0.06	0.35	1


P < 0.05: * (significant at the 0.05 level) ; P < 0.01: ** (significant at the 0.01 level); P < 0.001: *** (significant at the 0.001 level).

The correlation matrix presented in this study explores the relationships among variables associated with healthcare access. Notably, the variable “Difficulty to access to healthcare services (Y1)” exhibits a minimal positive correlation of 0.07 with the “Number of consultation (Y2),” implying a subtle tendency for increased difficulties in healthcare access to correspond with a slightly elevated frequency of consultations. The variables related to transportation, specifically “Mode of transportation (X2)” and “Number of modes of transportation (X2),” show positive correlations with both healthcare access variables, indicating a potential relationship between transportation factors and both the perceived difficulty of accessing healthcare services and the frequency of consultations. Of particular interest is the variable “Travel cost (X5),” which demonstrates substantial positive correlations with all variables, excluding the “Level of availability of transportation (X4)” where a noticeable negative association is observed.

The Pillai’s Trace statistic, is about 0.365, signifies a proportion of shared variance between the canonical variables, with an associated approximate F-statistic of 11.965 and degrees of freedom for hypothesis and error at 12 and 642, respectively. The Hotelling’s Trace statistic, showing a value of 0.46450, concurs with the Pillai’s Trace, demonstrating the robustness of the canonical correlations. The exact F-statistic for Wilks’ Lambda, standing at 12.157, demonstrates the statistical significance of the canonical correlation, with a Wilks’ Lambda of 0.663. These results, coupled with remarkably low p-values (0.000), lead to the rejection of the null hypothesis and implies a significant relationship between the transportation related barriers and access to healthcare facilities (see Table 3).

Examining the Wilks’s Lambda criterion, where Wilks’s λ is 0.66320 (F(12,11) = 0.66320, p = 0.000), we establish the statistical significance of the entire model across all functions. Investigating eigenvalues and canonical correlation results provides insight into the explained variability and the strength of associations among the sets of variables in the canonical correlation analysis. The first canonical correlation has an eigenvalue of 0.33516, contributing to 72.16% of the total variability in the set of variables related to Access to healthcare facilities. Meanwhile, the second canonical correlation, with an eigenvalue of 0.12934, adds an additional 27.84% of variability in the set of variables related to transportation related barriers. The cumulative percentage reaching 100% emphasizes that these two canonical correlations collectively capture all shared variability between the sets of variables. The canonical correlations, indicating relationship strength, are noteworthy. The first canonical correlation at 0.50 signifies a moderately strong linear relationship between the sets of variables. Its associated square correlation of 0.25 indicates that 25% of shared variability is explained in the set related to healthcare access. The second canonical correlation, with a value of 0.34 and a square correlation of 0.14, denotes a somewhat weaker, but still significant association in the set related to transportation related access.

SPSS extracted two canonical roots or dimensions for the model. The dimension reduction analysis tested the significance of each of the roots (see Table 5). The first test of significance showed that the full model across roots, 1 to 2 was significant **(Wilk’s λ = 0,66, F = 12.15, P < 0.01)**. The second test excluded the first root and test roots 2 showed it was also significant (Wilk’s λ = 0,66, F = 8.30, p = < 0.01). (See Table 4).

**Table 4:** Multivariate Tests of Significance.


TEST NAME	VALUE	APPROX.F	HYPOTHESIS DEGREES OF FREEDOM	ERROR DEGREES OF FREEDOM	SIGNIFICANCE OF F

Pillai’s	0.36555	11.96549	12	642	0.000

Hotelling’s	0.46450	13.34791	12	638	0.000

Wilks	0.66320	12.15701	12	640	0.000

Roys	0.25103				


*Note*: F statistic for WILKS ‘Lambda is exact.

**Table 5:** Eigenvalues and Canonical Correlation.


TEST NAME	EIGENVALUE	%	CUMULATIVE %	CANONICAL CORRELATION	SQUARE CORRELATION

**1**	**0.33516**	**72.16**	**72.16**	**0.50**	**0.25**

**2**	**0.12934**	**27.84**	**100**	**0.34**	**0.14**


The outcomes of the canonical correlation analysis reveal a noteworthy and statistically significant relationship between transportation barriers and access to healthcare facilities. Upon closer examination of Function 1 coefficients and canonical coefficient loadings, it becomes evident that the variable with the highest loading is “Percieved Difficulty to access healthcare services during the pandemic of Covid-19 (+0.98),” surpassing the loading of “Number of Medical visits during the past 12 months (+0.34).” In the context of the first canonical correlation, the relationship can be succinctly expressed through the canonical coefficients as follows (see Tables 6 and 7):

C1 = 0.98 × **Difficulty_to_access** + 0.34 × **Number_of_Medical_visits**

**Table 6:** Dimension Reduction Analysis.


ROOTS	WILKS L	F	HYPOTHESIS DEGREES OF FREEDOM	ERROR DEGREES OF FREEDOM	SIGNIFICANCE OF F

1 to 2	0.66320	12.15701	12	640	0.000

2 to 2	0.88548	8.30333	5	321	0.000


**Table 7:** Correlations Between Access to Healthcare Facilities and Canonical Variables.


ACCESS TO HEALTHCARE FACILITIES	FUNCTION 1	FUNCTION 2

Percieved Difficulty Access to healthcare services during the pandemic of Covid-19.	0.97796	–0.20879

Number of medical visits during the last 12 months.	0.33726	0.94141


According to Function 2, the canonical correlation analysis reveals a meaningful relationship between the covariate variables and the identified functions denoted as Function 1 and Function 2. Upon examining the coefficients of Function 1 and their corresponding canonical loadings, it becomes apparent that the variable exerting the most substantial influence is “Number of transportations **(+0.89)**,” surpassing the loadings of other covariates such as “Mode of Transportation **(+0.27**),” “Time Waiting for transportation **(–0.39)**,” “Availability of transportation **(–0.29)**,” “Affordability (Cost) **(+0.61)**,” and “Distance from nearest stop (+0.30).” Formally, the first canonical correlation can be expressed as (see Table 8):

C2 = 0.27 × **Mode_of_Transportation** + 0.89 × **Number_of_transportations** – 0.39 × **Time_Waiting_for_transportation** – 0.39 × **Availability_of_transportation** + 0.61 × **Affordability (Cost)** + 0.30 × **Distance_from nearest_stop**

**Table 8:** Correlations Between Transportation Barriers and Canonical Variables.


COVARIATE	FUNCTION 1	FUNCTION 2

Mode of Transportation	0.26784	0.63414

Number of Transportations	0.89116	0.05492

Time Waiting Transportation	–0.39614	0.02546

Availability of Transportation	–0.39671	0.34929

Affordability (Cost)	0.60584	0.33303

Distance from Nearest Stop	0.29650	–0.25829


This research sought to measure the link between transportation factors and access to healthcare services in the region of Guelmim Oued Noun (Morocco). The hypothesis which states that there is a significant correlation between transportation barriers and access to healthcare services was confirmed using empirical data from a regional survey. This study found that 56,10% of the sample studied have encountered many transportation barriers.

This study indicates that the cost of travel is the primary challenge faced by outpatients when attempting to access healthcare facilities. This finding is consistent with the socioeconomic status of the region, particularly in rural areas where poverty is more prevalent than in urban areas (6.9% compared to 5.10%). The results of this study align with those of another study conducted in Malawi, which aimed to examine the transportation challenges that affect access to healthcare facilities. The results demonstrate that the availability and affordability of transportation negatively impact access to healthcare facilities. This finding highlights the need for innovative solutions to address this barrier, particularly for vulnerable populations. Unfortunately, there is a lack of official statistics available about this specific population in order to understand their travel behavior towards healthcare destinations. Additionally, poor road conditions and inadequate transportation schedules were also reported as major barriers to accessing healthcare facilities.

Our findings also demonstrate that the number of transportation modes taken by outpatients and the transportation costs is highly contributing variables to transportation barriers which could be explained by the spatial dispersion of healthcare services across the region studied, more specifically in rural districts underserved by road networks. Further, outpatients who live in rural communities and seek specialized medical care should have at least two minimum modes of transportation to achieve the nearest provincial and regional healthcare services. The outpatient behavior towards the mode of travel depends also on the area of residence. Outpatients coming from rural communities faced more litany of challenges in getting formal transportation modes with unadopted time planning in comparison with those from urban areas. This result is in line with similar studies [49,50] where the mode of travel presents a decisive choice making for reaching healthcare facilities, especially in rural areas.

Additionally, the cost of transportation has been identified as a significant barrier to accessing healthcare, particularly for specialized medical treatments that are not available at primary or provincial healthcare centers. The financial burden placed on outpatients to reach regional or provincial healthcare centers can significantly impact their decision-making. Our study findings align with previous research that has also identified transportation barriers as a major obstacle to accessing healthcare facilities. Moreover, our study also revealed the difficulties faced in accessing healthcare facilities during the COVID-19 pandemic, as a result of various measures implemented by healthcare authorities to reduce the transmission of the virus in the community. The fear of contracting the virus and the lack of available transportation remain a significant challenge to accessing healthcare structures, especially for outpatients with chronic diseases and specific health needs. Similar results have been underlined in the perceived difficulty to access healthcare services examined in many studies [51,52] which reported, in common, the impact of the covid-19 outbreak on the access to the delivery of essential healthcare facilities.

To our knowledge, this is the first primary research study to systematically document the transportation barriers related to healthcare access in Morocco. In addition to practical and managerial implications, this study contributed to the extant literature by providing an understanding of the main factors contributing to transportation access and which can constitute a real challenge to access to healthcare services. From this perspective, a reflection on the necessary measures to be taken is highly recommended in order to overcome these difficulties, especially among vulnerable and socially excluded populations, such as women, children, and aged people. Also, the need for more detailed data about transportation barriers is highly required in order to understand the behaviors of outpatients and their different experiences. Implementing voucher schemes could have a positive impact on increasing the utilization of healthcare facilities, especially for pregnant women [53].

## Limitations

This study provides valuable insights and findings, however, there are a number of limitations that must be acknowledged. The use of a non-probabilistic sampling method limits the generalizability of findings to the larger population. The geographic scope of the study is also a limitation. The methodology of the study, which relied on subjective measures of access to healthcare, may have introduced bias. The study approached the issue of access to healthcare through an examination of various barriers, rather than isolating the specific impact of transportation on healthcare access. Additionally, the study was conducted during a specific period, and the negative effects of transportation on healthcare access may have been exacerbated by the regional-level measures and actions taken by healthcare authorities during this time.

## Conclusion

The present study aimed to address the empirical gap in understanding the relationship between transportation and access to healthcare facilities in the Moroccan context. Through the use of canonical correlation analysis, the study revealed a moderately significant and positive correlation between transportation and healthcare access. To the best of our knowledge, this is the first study to examine this relationship using this analytical method in the Moroccan context, with only official statistics previously reported on this topic in the 2018 National Health Strategy. Furthermore, this study serves as an important first step in assessing the variance explained by transportation in relation to healthcare access. The utilization of the canonical correlation method provides valuable statistical support for policymakers in transportation studies. In light of the results of this study, future research in this area is warranted. As a practical implication, it is suggested that more innovative strategies, such as Telehealth, be considered as a means to enhance access to specialized medical services among rural populations with transportation disadvantages. The implementation of e-health technology has emerged as a promising and transformative solution for outpatients across numerous African countries. This innovative strategy holds the potential to effectively address and mitigate transportation-related barriers, thereby contributing to the democratization of access to healthcare facilities. In regions where traditional healthcare infrastructure faces challenges, e-health technologies, including telemedicine and remote health monitoring, offer a promising solution and accessible alternative. By leveraging digital platforms, outpatients can remotely consult with healthcare professionals, receive medical advice, and even undergo virtual examinations. This not only diminishes the need for physical travel to healthcare facilities but also overcomes geographical constraints that may impede timely access to medical services.

## Footnotes

Chronic diseases and health promotion. 2012a Retrieved May 15, 2012, from http://www.cdc.gov/chronicdisease/overview/index.htm.
